# Comparing Characteristics of Endometrial Cancer in Women of South Asian and White Ethnicity in England

**DOI:** 10.3390/cancers13236123

**Published:** 2021-12-05

**Authors:** Seid Mohammed, Konstantinos Polymeros, Rochelle Wickham-Joseph, Iqra Luqman, Creana Charadva, Thomas Morris, Anna Collins, Shaun Barber, Kamlesh Khunti, Esther L. Moss

**Affiliations:** 1 Leicester Cancer Research Centre, University of Leicester, Leicester LE1 5WW, UK; seid.mohammed@leicester.ac.uk (S.M.); konstantinos.polymeros@nhs.net (K.P.); ac763@leicester.ac.uk (A.C.); 2 Leicester Clinical Trials Unit, University of Leicester, Leicester LE1 7RH, UK; sb766@leicester.ac.uk; 3 Department of Gynaecological Oncology, University Hospitals of Leicester, Leicester LE5 4PW, UK; r.wickham-joseph1@nhs.net (R.W.-J.); iqra.luqman@nhs.net (I.L.); creana.charadva@nhs.net (C.C.); thomasarthur.morris@nhs.net (T.M.); 4 Department of Health Sciences, University of Leicester, Leicester LE1 7RH, UK; 5 Diabetes Research Centre, University of Leicester, Leicester LE5 4PW, UK; kk22@leicester.ac.uk

**Keywords:** endometrial cancer, South Asian, ethnicity, diagnosis, risk-stratified, referral criteria

## Abstract

**Simple Summary:**

Endometrial cancer (EC) incidence is increasing many countries. Potential reasons for this change include increasing prevalence of risk factors, including high body mass index and diabetes, and falling hysterectomy rates for benign gynaecological conditions. Recent studies report increasing prevalence and differences in demography of EC in people from ethnic minority groups, particularly age at diagnosis and tumour subtype. This study was conducted to describe the demographic and EC tumour characteristics of White and South Asian ethnicity patients living in the same geographical region of England (Leicestershire), which is served by a single NHS hospital.

**Abstract:**

Differences in patient demographic and tumour characteristics between patients of South Asian and White ethnicity diagnosed with an endometrial cancer (EC) and currently living in England are not well described. We undertook a retrospective study of EC cases diagnosed at the University Hospitals of Leicester, UK. A total of 1884 cases were included, with 13% of the patients being of South Asian ethnicity. South Asian women were diagnosed at a significantly younger age (mean age of 60.3 years) compared to women of White ethnicity (mean age of 66.9 years) with a mean difference of 6.6 years (95% CI 5.1 to 8.1, *p* < 0.001). Rising body mass index (BMI) in the White patient group was significantly correlated with younger age at diagnosis (*p* < 0.001); however, this association was not seen in South Asian patients. A linear regression that adjusted for diabetes status, BMI, and the interaction terms of diabetes status with BMI and ethnicity with BMI, highlighted a younger age of diagnosis in South Asian patients with a BMI less than 45 kg/m^2^. The difference was greatest at lower BMIs for both non-diabetics and diabetics. Further investigation is needed to explain these differences and to determine their impact on suspected cancer referral criteria.

## 1. Introduction

Endometrial cancer (EC) is the most common gynaecological malignancy in the UK, with over 9700 new cases diagnosed each year [1]. The rising incidence of EC over the past two decades has been attributed to an increasing number of Type I tumours and linked with the rising prevalence of obesity [2]. Historically, EC is considered a cancer of older age, with only a small percentage of cases occurring in the pre-menopausal population, equating to 6.8% of women less than 50 years old at diagnosis [2]. This age distribution is reflected in the National Institute for Health and Care Excellence (NICE) referral guidelines for suspected EC [3], with an age of 55 years being the threshold referral age for a number of symptoms, and post-menopausal bleeding (PMB) being the only symptom recommended to prompt referral in patients under 55 years.

There is increasing evidence of clinical and pathological differences in EC between different populations, in particular, age at diagnosis [4], histological subtype [5], and survival [6,7]. Analysis of the EC molecular profile in The Cancer Genome Atlas (TCGA) database has shown differences in the frequency of commonly mutated EC genes in different ethnic groups [8], therefore emphasising the need for a more personalised approach to EC diagnosis and management.

In the 2013 National Cancer Intelligence Network (NCIN) report Outline of Uterine Cancer in the United Kingdom [9], Leicester City had the highest age-standardised incidence rate in England, 29.2/100,000, as compared to England as a whole at 19.6/100,000. Leicester is an ethnically diverse city with a high proportion of the population identifying as ‘Indian’ in the 2011 National census [10], in particular, of Gujarati Indian heritage [11]. EC data from the UK have not previously shown a difference in incidence by ethnicity [12]; however, differences have been reported in patients from different South Asian populations, with a 50% lower incidence noted in patients of Bangladeshi ethnicity, as compared to Indian, Pakistani, and White ethnicity [13].

Exploring cancer risk between patients from different ethnic populations is acknowledged to be challenging [14] and is often used as a proxy for investigating related genetic, environmental, and behavioural factors [15]. Cancer incidence soon after migration may change with increasing duration of time in the country of adoption and subsequent generations. For example, the incidence of uterine cancer amongst American Asian (Indian) women is increasing: the annual percentage change between 1990 and 2008 was 3.0 (95% CI 0.3–5.8) [16]. Differences in access to healthcare and medical insurance are also contributing factors in many countries.

The effect of risk factors for EC are reported to impact different populations to varying degrees; for example, a lower percentage rise in body mass index (BMI) is associated with a greater EC risk in Japanese American women compared to other ethnic groups [17]. It is not known whether the same effect is seen in South Asian populations, although the BMI thresholds for developing dysglycemia and dyslipidemia in the South Asian population are reported to be lower than in the White European population [18], and NICE advises consideration of lower BMI risk thresholds for Black, Asian, and other minority groups [19].

We investigated whether differences exist in the patient and EC characteristics of patients of South Asian and White ethnicity living in the same geographical region of England (Leicestershire), which is served by a single NHS hospital.

## 2. Methods

A retrospective review of all EC cases diagnosed at the University Hospitals of Leicester between 2003 and 2018 was undertaken. Permission for the study was given by the Hospital Audit Team (reference 7146) and the Office for Data Release, Public Health England (ODR1718_064). Cases were identified from the hospital pathology database and then checked with UK Cancer Registry data to ensure all cases were identified. Additional information was available for a subset of patients recruited between January 2016 and January 2020 to the ‘Identifying a high-risk profile in endometrial cancer’ study, which was granted ethical approval by the Yorkshire and the Humber-Leeds West Research Ethics Committee (15/YH/0510). Information was collated from the patients’ medical records on patient characteristics, including age at diagnosis, diabetes status (type I/II) and medication (including Metformin), ethnicity, measured body mass index (BMI), and tumour characteristics (histological subtype, grade, and stage). Information on menopausal status, age at menopause, parity, and waist/hip measurements was available for the subset of patients, included in the aforementioned study. Cases were staged using the FIGO 2009 classification and grouped according to the ESMO classification [20] into: (i) low-risk; (ii) intermediate and high-intermediate risk; and (iii) high-risk and advanced cases. Cases of EC recurrence and non-endometrial histology (stromal sarcoma, leiomyosarcoma, transitional cell carcinoma, and adenosarcoma) were excluded from the analysis. Patients’ self-designated ethnicity at the time of hospital registration was categorised into White (including British and Irish) and South Asian (including British Asian, Indian, Pakistani, and Bangladeshi). Patients from other ethnic groups were excluded from the analysis due to the small patient numbers.

### Statistical Analysis

Statistical analysis was performed using STATA (Version 16.0) (StataCorp LP, College Station, TX, USA). The association between categorical covariates and ethnicity was assessed using chi-square tests, with an option trend for the ordinal categorical covariates. A two-sided *p*-value was reported. Pearson’s correlation coefficient (r) was used to assess the relationship between the two continuous variables. A two sample T-test was used to compare the continuous outcome between the two groups. Standard deviation (SD) and mean difference (MD), alongside a two-sided 95% confidence interval (CI) and *p*-value, were reported. A linear regression was fitted, using a complete case analysis, to assess the effect of ethnicity on the age at diagnosis, adjusting for diabetes status and BMI, as well as statistically significant interactions between covariates in the model. Coefficients along with a two-sided 95% CI and *p*-value were reported. Metformin use was not included in the model to avoid the issue of multi-collinearity, as diabetes status and Metformin use are highly correlated. Interactions between covariates of the model were tested for using a likelihood ratio test with statistically significant (*p* < 0.05) interactions being added using stepwise forward selection. In all analyses, a *p*-value < 0.05 was considered statistically significant.

## 3. Results

A total of 1884 cases were included in the main analysis, of which 1633 (87%) were of White and 251 (13%) of South Asian ethnicity. There were significant differences in the characteristics of the two groups (Table 1).

**Figure 1:**
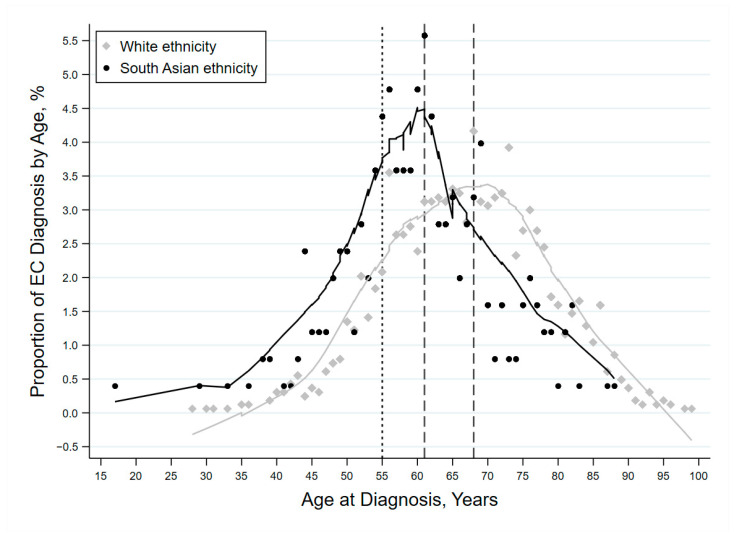
Distribution of age at diagnosis for women with endometrial cancer. The peak of each ethnicity represents the mode (vertical dashed line). The peak for the White ethnicity patients was at 68 years while for the South Asian ethnicity patients it was 61 years.

South Asian women were diagnosed at a significantly younger age (mean age 60.3 years; SD, 10.9) compared to women of White ethnicity (66.9 years; SD, 11.2) (MD = 6.6; 95% CI 5.1 to 8.1, *p* < 0.001) (Figure 1). Furthermore, the proportion of patients diagnosed below the age of 55 years was higher in patients of South Asian compared to White ethnicity (27.1% versus 13.2%), whereas the proportion of patients of White ethnicity that were over 70 years was significantly greater compared to the South Asian group (41.6% versus 17.5%) (*p* < 0.001) (Figure 1). The peak age for the White patient group was at 68 years, whereas for the South Asian patientsit was 61 years, a difference of 7 years.

The prevalence of type II diabetes (14.4% versus 35.5%) and Metformin use (8.4% versus 19.1%) was significantly higher in the South Asian group (*p* < 0.001).

The association of BMI with the age at diagnosis was different in the two groups, with rising BMI in the patients of White ethnicity significantly correlating with a younger age at diagnosis (r = −0.19, *p* < 0.001). The negative correlation between BMI and age at diagnosis remained significant in the White patient group with endometrioid Grade 1 and 2 cases (r = −0.20, *p* < 0.001 and r = −0.13, *p* = 0.02, respectively); however, the correlation was not significant in endometrioid Grade 3 and non-endometrioid cases (r = −0.11, *p* = 0.23 and r = −0.04, *p* = 0.680, respectively). The correlation between BMI and age at diagnosis was not seen in the South Asian patients(r = −0.03, *p* = 0.66). There was a negative correlation when subdividing the South Asian patients by diabetic status (r = −0.14), but this was not significant (*p* = 0.18). Significant differences were seen regarding the age at diagnosis in the BMI 25–29 kg/m^2^ and BMI 30–39 kg/m^2^ patients between the two groups, with a mean difference of 7.7 years (95% CI 4.2–11.1 years; *p* < 0.001) and 6.8 years (95% CI 4.6–8.9 years; *p* < 0.001), respectively. The difference was reduced when comparing the BMI > 40 kg/m^2^ patients, with only a 2.8-year difference (95% CI 0.1–5.6 years) (*p* = 0.042).

**Figure 2:**
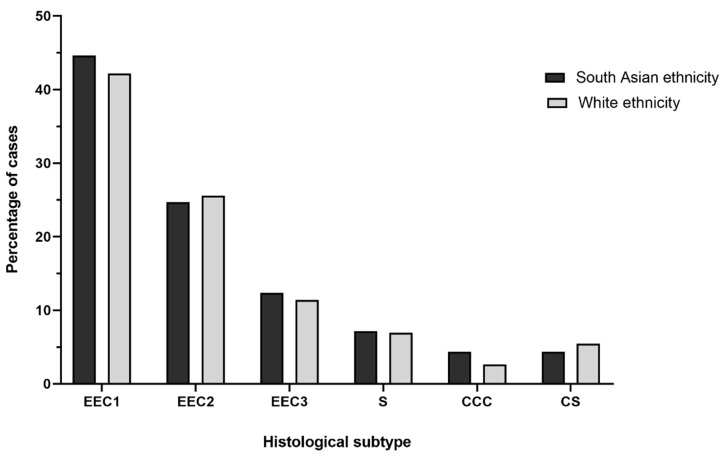
Histological subtypes of endometrial cancer in the White (*n* = 1535) and South Asian (*n* = 245) patient groups. Data presented are percentage of cases for the most common subtypes: EEC1 = endometrioid EC grade 1; EEC2 = endometrioid EC grade 2; EEC3 = endometrioid EC grade 3; S = serous; CCC = clear cell carcinoma; CS = carcinosarcoma.

Stage IA Grade 1 endometrioid EC was the most common diagnosis in both groups (Table 1). There was no difference in stage at diagnosis, risk classification, histological subtype (Figure 2), or tumour grade between the groups. 

The proportion of White ethnicity patients diagnosed in each of the time periods (2003–2008, 2009, and 2014–2018) was similar, with around a third of diagnoses occurring in each time period. On the other hand, the proportion of South Asian women diagnosed increased over the time periods: 21.5%, 33.5%, and 45.0%, respectively. This difference in year of diagnosis between White and South Asian women was statistically significant (*p* < 0.001) (Table 1).

A linear regression model for the outcome of age at diagnosis was fitted. The model included covariates for ethnicity (White or South Asian), BMI, and diabetes status. Using stepwise forward selection, interaction terms of BMI with diabetes status and BMI with ethnicity were selected for inclusion in the model. The likelihood ratio tests gave statistically significant results of *p* = 0.01 and *p* = 0.02, respectively, during the selection process. 

**Figure 3:**
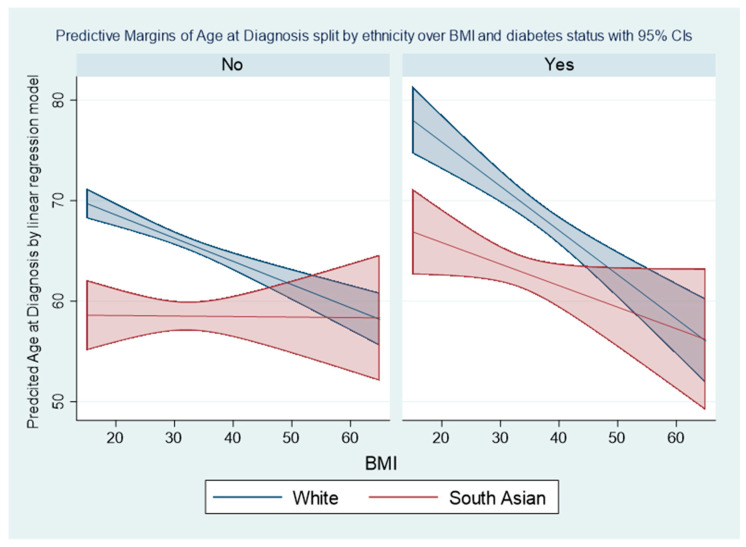
Predicted age of diagnosis with 95% confidence intervals for White and South Asian patients at different BMI values spilt by diabetes status (no/yes) from linear regression model. Model fitted based on 1344 patients with complete data for the variables in the model (920 non-diabetic White patients, 147 non-diabetic South Asian patients, 192 diabetic White patients, and 85 non-diabetic South Asian patients).

Figure 3 displays the difference between the predicted age of diagnosis for White compared to South Asian patients over differing BMI values for both non-diabetics and diabetics. For both non-diabetic and diabetic patients with a BMI less than 45, the age at diagnosis was younger for South Asian compared to White patients; however, this difference decreased as BMI increased, and for higher values of BMI the confidence intervals of the predicted age of diagnosis overlap. For both White and South Asian patients with a BMI less than 45, the age of diagnosis for diabetics was older than for non-diabetics. The linear regression model resulted in the predicted age of diagnosis decreasing as BMI increased in non-diabetic White patients, diabetic White patients, and diabetic South Asian patients; however, predicted age of diagnosis did not change as BMI increased for non-diabetic South Asian patients. The coefficients of this linear regression model are displayed in the supplementary material (Table S1).

Analysis of a subset of 216 cases (40 South Asian and 176 White ethnicity) confirmed a significant difference in the age at diagnosis (*p* = 0.0111) and the prevalence of type II diabetes (47.5% versus 18.2%; *p* < 0.0001) (Table S2). There was no difference in waist to hip ratios in the BMI categories for the two groups (Table S3). Parity was significantly higher in the South Asian group, with a median of three children (range 0–6) compared to two children (range 0–7) in the White patient group (*p* < 0.001). Three patients in each group reported a previous history of polycystic ovarian syndrome. The percentage of South Asian patients who were pre-menopausal at diagnosis was more than double that in the White ethnicity group, with 8 of 40 cases (20%) compared to 16 of 176 cases (9.1%), (*p* = 0.048). For the patients who were postmenopausal at the time of diagnosis, there was no difference in the age of menopause, with a median age of 51 years for both groups (*p* = 0.408).

## 4. Discussion

In this descriptive study, we observed significant differences in the characteristics of South Asian patients diagnosed with EC as compared to patients of White ethnicity living in the same geographical region and attending the same NHS hospital. Overall, South Asian patients were diagnosed with EC 6 years younger compared to women of White ethnicity. Our finding of a significantly younger age at diagnosis in an Asian population is not new [4,21]; however, this is the first study to describe the characteristics of co-located EC patients of South Asian and White ethnicity living in England. The age of menopause is reported to be lower in Asian as compared to Caucasian populations, at 49.1 years versus 51.4 years [22,23]. Our study showed no difference in the age of menopause between the two groups who were menopausal at EC diagnosis; however, it did identify that a greater proportion of South Asian patients were premenopausal at diagnosis (20%), as compared to the White ethnicity patients (9.1%). This finding has potential implications for accessing urgent referral pathways since criteria, such as NICE [3], focus on PMB and the threshold age of 55 years. Further research is needed to investigate the impact of age and ethnicity on the route to diagnosis, and the effect of a longer time to diagnosis on disease stage in premenopausal patients.

A possible reason for the younger age at diagnosis is Lynch syndrome [24], and although we were unable to perform this analysis in this study cohort, we have previously published on a matched cohort of patients of White/South Asian ethnicity and shown no difference in the presence of mismatch repair gene mutations between the two groups (20% versus 30%, *p* = 0.54) [25]. Lynch syndrome therefore does not appear to explain the age disparity. Differences in the mutational profile of common genes associated with EC have also been reported in the cohort of tumours from matched patients of White/South Asian ethnicity [25]; however, a study of a much larger population would be needed to investigate whether any particular mutation is associated with a younger age at diagnosis. The incidence of polycystic ovarian syndrome could be a reason for the age difference, with an incidence of 3/40 (7.5%) in the South Asian and 3/176 (1.7%) in the White ethnicity patients in the subgroup; again, further investigation in a larger population is needed to explore this potential association. Another reason could be due to the difference in age distribution of the White and South Asian populations in the UK, because the average age of the Asian/British and Asian/Indian population is lower than the White British population [10].

Our study showed that BMI in the White ethnicity group was inversely correlated with age at diagnosis in the Grade 1 and 2 endometrioid histology cases, as has been shown in other studies [26], but no significant association between age and BMI was seen in the non-endometrioid or Grade 3 endometrioid cases. Work by Gray et al. has reported differences in obesity cut-off points between a South Asian population resident in the UK, 21.5 kg/m^2^ for glycaemic factors (fasting glucose, 2 h glucose and HbA1c) and 23.9 kg/m^2^ for lipid factors (HDL cholesterol and triglycerides), as compared to 30 kg/m^2^ for a White European population. Therefore, although there was no difference in the BMI levels between the two groups in our study, given the large difference in BMI thresholds, a greater proportion of the South Asian patients would fall above these thresholds. There also appears to be an effect from diabetes in the South Asian patients. Studies have previously shown that the diagnosis of type II diabetes occurs 5–7 years earlier in South Asian patients as compared to White ethnicity groups, and at a lower BMI [27]. The impact of BMI and diabetes on EC risk and age at diagnosis therefore raises questions as to their impact on EC aetiology in the South Asian population, and whether primary prevention weight loss interventions may have an impact at the population level, particularly among South Asian groups. Investigation is needed to explore this area further.

The prevalence of type II diabetes was significantly higher in South Asian patient group, although the usage of Metformin appears to be lower than would be expected and could have been under-reported. Diabetes is well documented to be a risk factor for EC, although Metformin use is reported to be associated with a risk reduction of 13% in EC among patients with diabetes (relative risk 0.87) and improved survival in EC patients (hazard ratio 0.63) [28], including non-endometrioid EC [29]. Survival data were not available for our study cohort and further analysis is needed on larger populations to investigate the impact of diabetes and Metformin on the risk and survival of EC in patients of South Asian and White ethnicity.

Unlike previous studies, we have shown no difference in the stage at diagnosis between the South Asian and White patient groups. One reason for this may be that all patients were treated through the same NHS hospital and were not dependent on health insurance. There was also no difference in the histological subtypes between the groups, which would support our previous work showing that the three most commonly mutated genes in EC are the same in both the White and Asian TCGA populations [8].

We have also shown that the number of cases of EC diagnosed in South Asian patients in our geographical region is increasing. Further work is needed to determine whether this reflects a genuine change in age-standardised incidence rates within South Asian populations, or whether the rise is a result of an increasing, aging, or a demographically changing South Asian population within Leicestershire. In a previous study, we have shown that although EC patients of South Asian ethnicity had greater awareness of unscheduled vaginal bleeding being associated with a malignancy, they were less aware of the most common EC risk factors or the suspected cancer referral pathway, as compared to patients of White ethnicity [30]. Numerous barriers to help-seeking behaviour have been reported, in particular language [31], which could potentially delay an EC diagnosis; therefore, raising the awareness of EC and its most common presenting symptoms could facilitate investigations and encourage detection at the earliest point of contact.

### Limitations

Detailed information on menopausal status and age at menopause was only available for a subset of patients. There were also missing data points in the retrospective data, in particular BMI, which was not consistently documented in the earlier part of the cohort. The missing histology and stage data are due to all EC cases being included, as a result this information was not available for cases that did not undergo surgical management. Another limitation of this study is that potential confounder/covariates, such as cigarette smoking, alcohol consumption, dietary intake, physical activity, and family history of EC, were not available for study participants. The small sample size among the South Asian group could have had an effect on the estimates from statistical modelling and further investigations are needed in larger populations to confirm our findings.

## 5. Conclusions

We have described significant differences in demographic characteristics between co-located patients of South Asian and White ethnicity diagnosed with EC, in particular a younger age at diagnosis and a greater proportion of premenopausal cases seen in the South Asian patient group. Further investigation is needed to explain these differences and to determine their impact on suspected cancer referral criteria.

## Figures and Tables

**Table 1:** Patient and tumour characteristics.

Characteristics	Categories	White (*n* = 1633)	South Asian (*n* = 251)	*p*-Value *^≠^
Age at diagnosis in years	Mean (SD)	66.9 (11.2)	60.3 (10.9)	<0.001
	Median (IQR)	67 (59, 75)	60 (54, 67)	<0.001
BMI in Kg/m^2^	Mean (SD) ^	33.1 (8.9)	32.1 (7.4)	0.114
	Median (IQR) ^	32 (26, 39)	31 (27, 37)	0.443
Age group	Below 55	215 (13.2%)	68 (27.1%)	<0.001
	55–69	739 (45.3%)	139 (55.4%)	
	70 and above	679 (41.6%)	44 (17.5%)	
BMI group	Below 30	452 (27.7%)	90 (35.9%)	0.131 *^≠^*
	30–40	416 (25.5%)	101 (40.2%)	
	40 and above	255 (15.6%)	42 (16.7%)	
	Missing	510 (31.2%)	18 (7.2%)	
Type II Diabetes	No	1142 (70.0%)	151 (60.2%)	<0.001 *^≠^*
	Yes	233 (14.3%)	88 (35.2%)	
	Unknown	256 (15.7%)	11 (4.4%)	
Metformin use	No	1250 (91.6%)	191 (80.9%)	<0.001
	Yes	114 (8.4%)	45 (19.1%)	
Histological subtype	Endometrioid	1334 (81.7%)	209 (83.3%)	0.754 *^≠^*
	Non-Endometrioid	247 (15.1%)	41 (16.3%)	
	Unknown	52 (3.2%)	1 (0.4%)	
Stage of cancer	Stage I	1140 (69.8%)	195 (77.7%)	0.174 *^≠^*
	Stage II	155 (9.5%)	25 (10.0%)	
	Stage III and IV	198 (12.1%)	20 (8.0%)	
	Unknown	140 (8.6%)	11 (4.4%)	
Grade of cancer	Grade 1	693 (42.4%)	112 (44.6%)	0.792 *^≠^*
	Grade 2	424 (26.0%)	63 (25.1%)	
	Grade 3	428 (26.2%)	72 (28.7%)	
	Unknown	88 (5.4%)	4 (1.6%)	
Risk group	Low	595 (36.4%)	108 (43.0%)	0.441 *^≠^*
	Intermediate and H-intermediate	281 (17.2%)	43 (17.1%)	
	High/advanced	500 (30.6%)	75 (29.9%)	
	Unknown/NA	257 (15.7%)	25 (10.0%)	
Diagnosis year	2003–2008	540 (33.1%)	54 (21.5%)	<0.001
	2009–2013	553 (33.9%)	84 (33.5%)	
	2014–2018	540 (33.1%)	113 (45.0%)	

Data are *n* (%), unless otherwise stated. ^ Based on patients with available BMI data. * *p*-values < 0.05 were considered significant. **^≠^** *p*-values of the categorical variables were calculated using chi-square test excluding the unknown/missing. Abbreviations: BMI = Body Mass Index; SD = standard deviation; IQR = inter-quartile range.

## Data Availability

Data sharing is not applicable to this article due to ethical restrictions.

## Supplementary Materials

The following are available online at https://www.mdpi.com/article/10.3390/cancers13236123/s1, Table S1: Linear regression of age at diagnosis; Table S2: Characteristics of subgroup; Table S3: Descriptive statistics of waist:hip ratio by ethnicity for each BMI group.

## References

[1] Cancer Research UK Uterine Cancer Statistics. https://www.cancerresearchuk.org/health-professional/cancer-statistics/statistics-by-cancer-type/uterine-cancer/incidence.

[2] Evans T.R., Sany O., Pearmain P., Ganesan R., Blann A.D., Sundar S. (2011). Differential trends in the rising incidence of endometrial cancer by type: Data from a UK population-based registry from 1994 to 2006. Br. J. Cancer.

[3] National Institute for Health and Care Excellence Referral for Suspected Endometrial Cancer.

[4] Mahdi H., Schlick C.J., Kowk L.-L., Moslemi-Kebria M., Michener C. (2014). Endometrial cancer in Asian and American Indian/Alaskan Native women: Tumor characteristics, treatment and outcome compared to non-Hispanic white women. Gynecol. Oncol..

[5] Wright J.D., Fiorelli J., Schiff P.B., Burke W.M., Kansler A.L., Cohen C.J., Herzog T.J. (2009). Racial disparities for uterine corpus tumors. Cancer.

[6] Sherman M.E., Devesa S.S. (2003). Analysis of racial differences in incidence, survival, and mortality for malignant tumors of the uterine corpus. Cancer.

[7] Fader A.N., Habermann E., Hanson K.T., Lin J.F., Grendys E.C., Dowdy S.C. (2016). Disparities in treatment and survival for women with endometrial cancer: A contemporary national cancer database registry analysis. Gynecol. Oncol..

[8] Guttery D., Blighe K., Polymeros K., Symonds R.P., Macip S., Moss E. (2018). Racial differences in endometrial cancer molecular portraits in The Cancer Genome Atlas. Oncotarget.

[9] NCIN (2013). Outline of Uterine Cancer in the United Kingdom: Incidence, Mortality and Survival.

[10] Office for National Statistics 2011 Census. https://www.ons.gov.uk/peoplepopulationandcommunity/culturalidentity/ethnicity/articles/ethnicityandnationalidentityinenglandandwales/2012-12-11#animated-youtube-video.

[11] Lord K., Ibrahim K., Kumar S., Rudd N., Mitchell A., Symonds P. (2012). Measuring Trust in Healthcare Professionals—A Study of Ethnically Diverse UK Cancer Patients. Clin. Oncol..

[12] National Cancer Intelligence Network and Cancer Research UK (2009). Cancer Incidence and Survival by Major Ethnic Group, England 2002–2006. http://ncin.org.uk/search/cancer+and+incidence+by+major+ethnic+group+england+2002+2006.

[13] Shirley M.H., Barnes I., Sayeed S., Finlayson A., Ali R. (2014). Incidence of breast and gynaecological cancers by ethnic group in England, 2001–2007: A descriptive study. BMC Cancer.

[14] Yudell M., Roberts D., DeSalle R., Tishkoff S. (2016). Taking race out of human genetics. Science.

[15] Sankar P., Cho M.K. (2002). Toward a New Vocabulary of Human Genetic Variation. Science.

[16] Gomez S.L., Noone A.-M., Lichtensztajn D.Y., Scoppa S., Gibson J.T., Liu L., Morris C., Kwong S., Fish K., Wilkens L.R. (2013). Cancer Incidence Trends Among Asian American Populations in the United States, 1990–2008. J. Natl. Cancer Inst..

[17] Park S.L., Goodman M.T., Zhang Z.-F., Kolonel L.N., Henderson B.E., Setiawan V. (2009). Body size, adult BMI gain and endometrial cancer risk: The multiethnic cohort. Int. J. Cancer.

[18] Gray L., Yates T., Davies M., Brady E., Webb D.R., Sattar N., Khunti K. (2011). Defining Obesity Cut-Off Points for Migrant South Asians. PLoS ONE.

[19] NICE. https://www.nice.org.uk/News/Article/consider-lower-bmi-risk-thresholds-for-people-from-black-asian-and-minority-groups.

[20] Colombo N., Creutzberg C., Amant F., Bosse T., Martín A.G., Ledermann J., Marth C., Nout R.A., Querleu D., Mirza M.R. (2016). ESMO-ESGO-ESTRO Consensus Conference on Endometrial Cancer: Diagnosis, Treatment and Follow-up. Int. J. Gynecol. Cancer.

[21] Zhang M.M., Cheung M.K., Osann K., Lee M.M., Gomez S.S.L., Whittemore A.S., Husain A., Teng N.N., Chan J.K. (2006). Improved Survival of Asians With Corpus Cancer Compared With Whites. Obstet. Gynecol..

[22] Chim H., Tan B.H.I., Ang C.C., Chew E.M.D., Chong Y.S., Saw S.M. (2002). The prevalence of menopausal symptoms in a community in Singapore. Maturitas.

[23] Boulet M., Oddens B., Lehert P., Vemer H., Visser A. (1994). Climacteric and menopause in seven south-east Asian countries. Maturitas.

[24] Broaddus R.R., Lynch H.T., Chen L.-M., Daniels M., Conrad P., Munsell M.F., White K.G., Luthra R., Lu K.H. (2005). Pathologic features of endometrial carcinoma associated with HNPCC. Cancer.

[25] Polymeros K., Guttery D.S., Hew R., Bishop R., Stannard E., Macip S., Symonds P., Moss E. (2020). Differences in the molecular profile of endometrial cancers from British White and British South Asian women. PLoS ONE.

[26] Nevadunsky N.S., Van Arsdale A., Strickler H.D., Moadel A., Kaur G., Levitt J., Girda E., Goldfinger M., Goldberg G.L., Einstein M.H. (2014). Obesity and Age at Diagnosis of Endometrial Cancer. Obstet. Gynecol..

[27] Paul S.K., Adjah E.S.O., Samanta M., Patel K., Bellary S., Hanif W., Khunti K. (2017). Comparison of body mass index at diagnosis of diabetes in a multiethnic population: A case-control study with matched non-diabetic controls. Diabetes Obes. Metab..

[28] Tang Y.-L., Zhu L.-Y., Li Y., Yu J., Wang J., Zeng X.-X., Hu K.-X., Liu J.-Y., Xu J.-X. (2017). Metformin Use Is Associated with Reduced Incidence and Improved Survival of Endometrial Cancer: A Meta-Analysis. BioMed Res. Int..

[29] Nevadunsky N.S., Van Arsdale A., Strickler H.D., Moadel A., Kaur G., Frimer M., Conroy E., Goldberg G.L., Einstein M.H. (2014). Metformin use and endometrial cancer survival. Gynecol. Oncol..

[30] Kumarakulasingam P., McDermott H., Boutler L., Patel N., Tincello D., Moss E. (2018). Knowledge of the risk factors and symptoms associated with endometrial cancer in British South Asian and British White women. Eur. J. Obstet. Gynecol. Reprod. Biol..

[31] Kolar S.K., Wheldon C., Hernandez N.D., Young L., Romero-Daza N., Daley E.M. (2014). Human Papillomavirus Vaccine Knowledge and Attitudes, Preventative Health Behaviors, and Medical Mistrust Among a Racially and Ethnically Diverse Sample of College Women. J. Racial Ethn. Health Disparities.

