# Supplementary material for: Comparing Characteristics of Endometrial Cancer in Women of South Asian and White Ethnicity in England
Source: Cancers (Basel). 2021 Dec 5;13(23):6123. doi: 10.3390/cancers13236123 (PMC8657185; doi:10.3390/cancers13236123)
Supplement: Supplementary file 1 [file cancers-13-06123-s001.zip › cancers-1240864-supplementary.pdf]

Table S1. Linear regression of Age at Diagnosis

Term	Coefficient (β)	95% CI for β	P Value
Ethnicity (South Asian)	−14.51	−20.98 to −8.04	<0.001
Diabetes Status (Yes)	11.44	5.66 to 17.22	<0.001
BMI (kg/m²)	−0.23	−0.31 to −0.15	<0.001
Ethnicity (South Asian)* BMI (kg/m²)	0.23	0.03 to 0.42	0.023
Diabetes Status (Yes)* BMI (kg/m²)	−0.21	−0.37 to −0.05	0.009
Constant	73.19	70.57 to 75.80	<0.001

Table S2. Characteristics of subgroup, n = 216.

Characteristics	Categories	White Ethnicity (N = 176)	South Asian Ethnicity (N = 40)	*p-Value* *≠
Age at diagnosis in years	Mean (SD)	64.8 (10.6)	59.9 (10.3)	0.008
	Median (IQR)	64.5 (57, 73)	59.9 (54.5, 67)	0.011
BMI in Kg/m²	Mean (SD) ^	36.6 (9.6)	33.9 (7.3)	0.087
	Median (IQR) ^	32.3 (29, 39)	35.7 (30, 42)	0.095
Age group	Below 55	27 (15.3%)	10 (25.0%)	0.119
	55–69	82 (46.6%)	21 (52.5%)	
	70 and above	67 (38.1%)	9 (22.5%)	
BMI group	Below 30	41 (23.3%)	13 (32.5%)	0.417 ≠
	30–40	80 (45.5%)	18 (45.0%)	
	40 and above	53 (30.1%)	9 (22.5%)	
	Missing	2 (1.1%)	0 (0%)	
Type II diabetes	No	146 (83.0%)	21 (52.5%)	< 0.001
	Yes	30 (17.0%)	19 (47.5%)	
Metformin use	No	156 (88.6%)	29 (72.5%)	0.009
	Yes	20 (11.4%)	11 (27.5%)	
Histological subtype	Endometrioid	150 (85.2%)	33 (82.5%)	0.632
	Non-Endometrioid	26 (14.8%)	7 (17.5%)	
Stage of cancer	Stage I	153 (86.9%)	36 (90.0%)	0.611≠
	Stage II	9 (5.1%)	3 (7.5%)	
	Stage III & IV	10 (5.7%)	1 (2.5%)	
	Unknown/Missing	4 (2.3%)	0 (0%)	
Grade of cancer	Grade 1	112 (63.6%)	21 (52.5%)	0.190≠
	Grade 2	24 (13.6%)	10 (25.0%)	
	Grade 3	37 (21.0%)	9 (22.5%)	
	Unknown	3 (1.7%)	0 (0%)	
Risk group	Low	113 (64.2%)	19 (47.5%)	0.008≠
	Intermediate & H-intermediate	27 (15.3%)	15 (37.5%)	
	High and advanced	32 (32.0%)	6 (15.0%)	
	Unknown/not applicable	4 (2.3%)	0 (0%)	

Data are n (%), unless otherwise stated.

^ Based on patients with available BMI data.

** p-value < 0.05 were considered significant*

≠ P-value of the categorical variables were calculated using chi-square test excluding the unknown/missing

Abbreviations: BMI = Body Mass Index; SD = standard deviation; IQR = Inter-quartile range

Table S3. Descriptive statistics of waist:hip ratio by ethnicity for each BMI group.

Measurements	BMI Group	South Asian Ethnicity (n = 22)		White Ethnicity (n = 77)	
		N	Mean (SD)	N	Mean (SD)
Waist (cm)	BMI < 30	7	91.4 (11.2)	16	92.6 (11.2)
	BMI 30–39	11	108.5 (11.4)	35	110.9 (8.9)
	BMI 40–49	3	123.7 (8.6)	17	129.0 (11.1)
	BMI ≥ 50	1	130 (0.0)	9	132 (11.5)
Hip (cm)	BMI < 30	7	101 (6.2)	16	104.2 (8.0)
	BMI 30–39	11	121.6 (9.7)	35	121.1 (9.8)
	BMI 40–49	3	133.7 (9.0)	17	143.0 (7.2)
	BMI ≥ 50	1	141 (0.0)	9	153.7 (9.3)
Waist:Hip ratio	BMI < 30	7	0.90 (0.07)	16	0.89 (0.08)
	BMI 30–39	11	0.89 (0.06)	35	0.92 (0.05)
	BMI 40–49	3	0.93 (0.03)	17	0.89 (0.06)
	BMI ≥ 50	1	0.92 (0.0)	9	0.86 (0.07)

Abbreviations: N = number of patients; BMI = Body Mass Index; SD = standard deviation
